# Burning Mouth Syndrome From Statin Use: A Case Study

**DOI:** 10.7759/cureus.75223

**Published:** 2024-12-06

**Authors:** Kaitlyn M Blackburn, Jeffrey Esper

**Affiliations:** 1 Neurology, University of Pittsburgh Medical Center Hamot, Erie, USA

**Keywords:** acquired neuropathy, burning, small fiber neuropathy, statin, statin reaction

## Abstract

Statins are one of the most commonly prescribed medications in America. They are known for their ability to decrease cholesterol. Although generally well-tolerated, they are known to cause a variety of moderate side effects. Herein, we report on a rarely reported side effect of statin-induced neuropathy. A 35-year-old male with type IV hyperlipoproteinemia presented to the neurology outpatient clinic with complaints of tongue burning after taking a statin for just seven days. After being placed on Atorvastatin 20 mg daily, he developed dysesthesias in his tongue and mouth. No other prescribed or over-the-counter medications were being taken at the time. A detailed neurological examination was conducted and was found to be normal, besides dysesthesias of the tongue. Atorvastatin was discontinued, and the burning resolved within three weeks. Burning mouth syndrome (BMS) is a disorder that causes painful dysesthesias of the tongue thought to be caused by small fiber neuropathy. Small fiber neuropathy affects sensory and autonomic small fibers, resulting in both sensory and autonomic symptoms. Sensory symptoms can include burning, tingling, and stabbing discomfort. Though there are many causes of small fiber neuropathy, this case is rare in the fact that it only involves the patient’s tongue.

## Introduction

Worldwide, nearly 25% of the population over the age of 65 is prescribed a statin [1]. Statins are indicated in many neurological, cardiovascular, and endocrine disorders. They are most commonly used in secondary prevention of stroke and heart attack. In particular, atorvastatin is a moderate to high-intensity statin. Statins have anti-inflammatory, antioxidant, and immunomodulatory properties and are commonly used in multiple conditions [2]. While statins are beneficial in many diseases, they have been known to cause a variety of adverse events. Patients taking only statins usually tolerate them well, but in combination with other medications, the risk of adverse events increases. The most encountered side effect of statin usage is myalgia, which occurs in 1%-10% [3].

## Case presentation

A 35-year-old male presented to an outpatient neurology clinic for evaluation of a burning sensation in his tongue. Medical history included benign skin lesions, inguinal hernia, and type IV hyperlipoproteinemia. The patient had no history of alcohol or tobacco use. His family history was significant for hyperlipoproteinemia, Parkinson’s disease, malignant melanoma, and peptic ulcer disease. Notable lab values included a triglyceride level of 543 mg/dL (normal under 150 mg/dL) and a high-density lipoprotein (HDL) of 22 mg/dL (normal above 40 mg/dL). Due to the high triglyceride levels, he was recommended numerous medications in the past. The patient had been treated for this in the past with niacin, though this is not a first-line treatment. This was later discontinued due to intolerable pruritus, which resolved with cessation. He was then started on Fenofibrate 145 mg. He subsequently developed proximal weakness, with creatine phosphokinase (CPK) levels of over 800 IU/L. This treatment was also discontinued. Several months later, the patient was started on Atorvastatin 20 mg. Within seven days, the patient developed a burning sensation in his tongue and mouth. His taste remained intact. He described the feeling as if he ate too many chili peppers. He was not on any other medications at that time. 

Neurological examination revealed tongue dysesthesias. He had no sensory disturbances in his extremities, and vibration and position senses were intact. Reflexes were normal throughout. Muscle strength was equal throughout. The remainder of his neurological examination was normal. 

Lab work completed at this time included fasting glucose, vitamin B-12 levels, methylmalonic acid level, immunofixation study, and Sjogren’s panel. These results all returned normal. Atorvastatin was discontinued. It took three weeks for the symptoms to completely resolve.

## Discussion

The nerves of the peripheral nervous system contain both large and small fibers. Large fibers carry messages from the brain responsible for movement, vibration, and joint position. Small fibers transmit information about pain, temperature, and visceral functions, like blood pressure and heart rate. It is seen that those with large fiber neuropathy experience loss of joint position, vibration sense, and sensory ataxia [4]. Other patients have reported symptoms of acral burning pain, paresthesias, dysesthesias, and pruritis [5]. 

Though rare, statins have been found to cause large fiber neuropathy, leading to muscle wasting, weakness, and areflexia [6]. Less common are small fiber neuropathies, which present with sensations of burning or coldness. Prior reports have documented four cases of small fiber neuropathy where statins were associated with sensory symptoms, abnormal sympathetic skin responses [7], and abnormal reflexes [8]. 

Prior research has determined that statin use has been associated with sensory and autonomic fiber degeneration. Biopsy revealed autonomic and dorsal root ganglion lesions, indicating a non-length-dependent process. While biopsy-determined small fiber neuropathy has been associated with statin use, there have been no documented cases involving sensory changes of the tongue. 

The patient in our study had no prior documented neuropathy. He had no known autoimmune disorder, paraneoplastic syndrome, infection, alcohol abuse, or any disability that might have contributed to his symptoms. He was not taking any other medications at the time of symptom onset. The patient's symptoms resolved within three weeks after discontinuing atorvastatin. While there have been documented cases of statin-induced small fiber neuropathy, BMS has not been described in this scenario. Thus, we have exposed another side effect of statin use. Due to the necessity of statins, especially atorvastatin, in almost every specialty in medicine, it is vital to be aware of potential rare side effects of these medications.

## Conclusions

In conclusion, it is important to be aware of all typical and atypical medication side effects and reactions. This particular patient presented with rare symptoms that were ultimately related to the use of a statin medication, though this reaction had never been documented in literature. Statin medications are essential in the treatment and prevention of multiple vascular disorders and are commonly prescribed by physicians in all areas of expertise. Therefore, it is extremely important for all prescribers, regardless of their fields of expertise, to be familiar with the potential side effects of statins. 
